# Intrathoracic Injection of Iodine in Tuberculosis

**Published:** 1913-11

**Authors:** 


Intrathoracic Injection of Iodine in Tuberculosis. D'Amico, *Lancet,* March 29, 1913, advises the following solution: Iodoform 1., Camphor 2, Guaiacol 5, Essence of peppermint 2., Olive oil 20. 1 c.c. up to 4-6 c.c. for cavities, is injected through a long, slender, plantinoiridium needle into the pulmonary lesion, as nearly as it can be determined. 10 injections usually suffice for children, 20-60 for adults. Improvement amounting to cure is claimed, but ordinary hygienic treatment was also carried out. The cardiac area and large blood vessels should be avoided. The injections are made usually in the second to the sixth intercostal spaces, the apices being reached from behind, above the scapula. (Note. A similar treatment was in vogue about twenty-five years ago. We happened to witness an injection which resulted in fatal pneumothorax, the needle having accidentally punctured an emphysematous portion of lung so friable that the tissues did not close after it. While one should not be unduly influenced by a single unfortunate result, an experience of this sort naturally prejudiced us in favor of medication through the air passages, blood and lymphatics.)

